# Postoperative Pain Control for Total Knee Arthroplasty: Continuous Femoral Nerve Block Versus Intravenous Patient Controlled Analgesia

**DOI:** 10.5812/aapm.3404

**Published:** 2012-04-01

**Authors:** Rui Min Lee, John Boon Lim Tey, Nicholas Hai Liang Chua

**Affiliations:** 1 Department of Anesthesiology, Intensive Care and Pain Medicine, Tan Tock Seng Hospital, Singapore

**Keywords:** Nerve Block, Analgesia, Patient Controlled, Arthroplasty, Replacement, Knee

## Abstract

**Background:**

Pain after total knee arthroplasty is severe and impacts functional recovery.

**Objectives:**

We performed a retrospective study, comparing conventional patient control analgesia (PCA) modalities versus continuous femoral nerve blockade (CFNB) for 1582 post-TKA (total knee arthroplasty) patients.

**Patients and Methods:**

Using our electronic acute pain service (APS) database, we reviewed the data of 579 patients who had received CFNBs compared with 1003 patients with intravenous PCA over 4 years.

**Results:**

Our results show that the incidence of a severe pain episode was higher in the PCA compared with the CFNB group. Lower pain scores were observed in the CFNB group compared with the PCA group from postoperative day (POD) 1 to 3, primarily due to lower rest pain scores in the CFNB group.

**Conclusions:**

Our study shows that there is improvement in pain scores, at rest and on movement, as well as a reduction in incidence of severe pain, in patients who receive CFNB versus those who receive intravenous PCA.

## 1. Background

Severe postoperative pain is a major complaint in patients who have undergone total knee arthroplasty (TKA). In addition, post-TKA pain directly impacts postoperative physiotherapy and mobilization, which can result in stiffness and poor joint function (1-3). Post-TKA pain peaks up to 48 hours after surgery (4).

Effective postoperative pain control is important, especially with the initiation of physiotherapy and early ambulation, which hastens recovery and reduces hospital length of stay (5). The risk of postoperative complications, such as venous thromboembolism and nosocomial infections (6), has also been shown to decrease with early mobilization.

Pain control modalities for post-TKA include intravenous patient-controlled analgesia (PCA), peripheral nerve blockade, and continuous epidural analgesic techniques. All methods have been shown to be efficacious in relieving postoperative pain. However, conventional techniques that use intravenous PCA with morphine and fentanyl are associated with side effects, such as respiratory depression, sedation, pruritus, nausea and vomiting, hypotension, constipation, and urinary retention (7); epidural analgesia is associated with hypotension and bilateral motor blockade if local anesthetics are used (8, 9).

## 2. Objectives

We performed a retrospective study, comparing conventional PCA modalities versus continuous femoral nerve blockade (CFNB) for 1582 post-TKA patients.

## 3. Patients and Methods

We reviewed the electronic acute pain service (e-APS) records of patients who underwent total knee arthroplasty between May 2006 and December 2010. Patients were included if they had received a CFNB via catheter or intravenous PCA with opioids. Intravenous opioid was not used in the postoperative period in the CFNB group.

Both groups were provided with adjunct analgesics, such as paracetamol and nonsteroidal anti-inflammatory drugs (NSAIDS) namely, etoricoxib, celecoxib, and ibuprofen depending on the patient’s clinical condition. Oral opioids, such as tramadol and oxycodone, were given for breakthrough pain in the CFNB group. Data on catheter placement methods (i.e., ultrasonography-guided or nerve stimulator-assisted), sensory stimulation parameters, and volume of initial LA bolus were not available in the electronic database.

The main outcome measures were:

1. Incidence of severe pain [defined as visual analog scale (VAS) ≥ 6]
2. VAS (0 = no pain; 10 = worst possible pain) for rest and dynamic pain on POD 1 to 3. The use of adjunct analgesics, such as oral paracetamol and NSAIDS, as well as significant adverse reactions (respiratory depression and hypotension) from PCA or CFNB, was also compared.

Sample means and standard deviations (SD) were applied to continuous baseline characteristics data. Frequency counts were used to summarize categorical data. The normal distribution of samples was tested by the degree of skewness and kurtosis for all baseline characteristics and measured parameters and was subsequently compared by independent *t*-test, for which equal variances were assumed only when *P* > 0.05 by Levene’s test for equality of variances. The categorical baseline data and incidence of side effects were compared by chi-square test.

Due to the multiple daily pain scores that were compared, multivariate testing using the general linear model was performed for VAS at rest and during movement from Days 1 to 3 for the PCA versus CFNB group. Post hoc analysis (Tukey’s HSD) was applied to interactions that were statistically significant. All analyses were performed using the SPSS for Windows (version 16.0). The significance level was set to *P* ≤ 0.05.

## 4. Results

The 1582 patients who underwent TKA from May 2006 to December 2010 received PCA (n = 1003) or CFNB (n = 579) exclusively. The PCA and CFNB groups were similar with regard to age, gender, and usage of adjunct analgesics, such as paracetamol and NSAIDs (Table 1).

The incidence of severe pain (VAS ≥ 6) from post operative day (POD) 1-3 was higher in the PCA group compared with the CFNB group (PCA = 69.1% and CFNB = 32.3%; *P* = 0.003). Individual effects of time (F = 95.7, *P* < 0.001) and VAS (F = 6.7, *P* < 0.001) were significant between the PCA and CFNB groups. The following interactions were significant: time-group (F = 27.6, *P* < 0.001); VAS-group (F = 113.7, *P* < 0.001), and time-VAS (F = 20.8, *P* < 0.001). The time-VAS-group interaction was not significant (*P* > 0.05) (Table 2).

For the time-group interaction, the post hoc analysis revealed:

1. Lower pain scores in the CFNB group compared with the PCA group on POD 2 compared with POD 1 (*P* = 0.048).
2. Lower pain scores in the CFNB group compared with the PCA group on POD 3 versus 2 (*P* < 0.001).

For the VAS-group interaction, the post hoc analysis showed lower rest pain scores compared with dynamic pain scores in the CFNB versus PCA group (*P* < 0.001).

In summary, the incidence of a severe pain episode was higher in the PCA group compared with the CFNB group. By multivariate testing, pain scores in both groups were lower on POD 2 versus POD 1 and on POD 3 compared with POD 2. This change was significantly lower in the CFNB group versus the PCA group from POD 1 to 3. Overall, lower rest pain scores were observed significantly lower in the CFNB group versus the PCA group. There was 1 incident of hypotension and 1 incident of respiratory depression in the PCA group but no documented adverse events in the CFNB group.

**Table 1.** Patient Characteristics at Baseline

	PCA ^a^, (n = 1003)	FNB ^a^, (n = 579)
Age, y, Mean ± SD	65.57 ± 8.5	65.81 ± 8.5
Gender, No. (%)		
Male	206 (20.5)	112 (19.3)
Female	797 (79.5)	467 (80.7)
Paracetamol usage, No. (%)	977 (97.4)	571 (98.6)
NSAIDs ^a^ usage, No. (%)	784 (78.2)	458 (79.1)

^a^ Abbreviations: FNB, femoral nerve block; NSAIDs, non-steroidal anti-inflammatory drugs; PCA, patient controlled analgesia

**Table 2.** VAS ^a^ Scores on POD ^a^ 1-3

	PCA ^a^ Group		CFNB ^a^ Group	
	Rest Pain	Dynamic Pain	Rest Pain	Dynamic Pain
POD 1, Mean ± SD	1.07 ± 1.69	5.11 ± 1.83	0.43 ± 1.12	3.47 ± 2.21
POD 2, Mean ± SD	0.77 ± 1.31	4.92 ± 1.85	0.33 ± 0.87	3.52 ± 1.90
POD 3, Mean ± SD	0.26 ± 0.84	3.81 ± 1.73	0.24 ± 0.72	3.06 ± 1.91

^a^ Abbreviations: CFNB, continuous femoral nerve block; PCA, patient controlled analgesia; POD, postoperative day; VAS, visual analog scale

## 5. Discussion

Our study shows that there is improvement in pain scores, at rest and on movement, as well as a reduction in the incidence of severe pain in patients who receive CFNB versus those who receive intravenous PCA. There were no documented adverse events in the CFNB group, whereas an incident of hypotension and respiratory depression was noted in the PCA group. We did not record common side effects, such as pruritus, nausea, and vomiting. To our knowledge, this is the largest single-center retrospective study of CFNB alone versus PCA.

Severe pain after total knee arthroplasty has been shown to impact functional recovery (10). Various studies have demonstrated the effectiveness of femoral nerve blocks in reducing pain post-total knee arthroplasty compared with intravenous PCA (3, 7, 8, 11-17). A systematic review of 112 studies between 1996 and 2005 by the PROSPECT working group supports the use of femoral nerve blocks for postoperative analgesia for primary TKA, based on the reduction in pain scores and supplemental analgesia (10). Of the 112 studies, 5 investigated the use of continuous infusion femoral nerve block versus placebo (8, 18-21), all of which noted reduced pain scores at rest and with motion at 24 and 48 hours with CFNB.

Similarly, a recent meta-analysis by Paul *et al*. (6) found that single-shot and continuous FNB was superior (lower opioid consumption) to PCA alone. In particular, continuous FNB with intravenous PCA was clearly superior to PCA alone with regard to reduced morphine consumption at 24 and 48 hours, pain scores, and nausea. Currently, there is a paucity of evidence to support the use of single-shot femoral nerve block as opposed to continuous nerve block technique. In 2 studies (20, 22), however, the authors showed significant improvements in analgesic use at rest and on movement, as well as opioid-sparing benefits, with single shot nerve blocks.

Because our report was a retrospective case review, there are inherent limitations. The data were obtained from an electronic database; thus, details, such as the method of insertion of the femoral nerve catheter, mode of anesthesia, and surgical technique, were unavailable. For future studies, it might be useful to perform continuous FNB, which allows one to control the concentration of the local anesthetic dose, type of local anesthetic, and infusion regimen, which preserves the quadriceps function better, resulting in earlier mobilization (23).

In total knee arthroplasty, postoperative pain control is imperative in facilitating physical therapy and rehabilitation. Our study demonstrated a significant reduction in the incidence of severe pain with CFNB versus PCA, with lower pain scores to Day 3 post-TKA. In addition, we did not observe any adverse effects from CFNB, such as hypotension and respiratory depression. Thus, CFNB is a safe alternative for post-TKA pain control.
